# Predictors Affecting Diabetes Related Distress among Diabetes Patients

**DOI:** 10.21315/mjms2022.29.2.9

**Published:** 2022-04-21

**Authors:** Jia Yii LIEW, Divya VANOH

**Affiliations:** Dietetics Programme, School of Health Sciences, Universiti Sains Malaysia, Kelantan, Malaysia

**Keywords:** diabetes related distress, diabetes, hand grip strength, smoking, marital status

## Abstract

**Background:**

Diabetics experienced distress due to the disease. This distress may impact the quality of life and adherence to treatment by the diabetics. Thus, this study is aimed at identifying the factors affecting diabetes-related distress (DRD) among diabetic patients.

**Methods:**

A total of 100 diabetic subjects aged 18 years old and above were recruited. Data on socio-demographic data, anthropometry, hand grip strength (HGS) and body fat percentage were obtained. DRD was assessed using the Malay version of the 17-item diabetes distress scale (MDDS-17) questionnaire. Multiple linear regression was employed to identify the predictors of DRD and the significance value was set at *P* < 0.05.

**Results:**

The majority of the subjects had low DRD (93%). Univariate analysis revealed that higher DRD scores were correlated with being single, never exercising and having lower education level, body weight, body fat percentage, body mass index (BMI) as well as HGS (*P* < 0.05). However, further multivariate regression analysis revealed that only smoking and being single/divorced/widow were the predictors of DRD.

**Conclusion:**

Predictors of DRD in this study were smoking and being either single/divorced/widow. These factors must be taken into consideration during the medical management of diabetics in order to ensure more holistic management of the disease and the distress it caused.

## Introduction

With diabetes becoming a major public health concern and with more than 180 million diabetics worldwide, it is expected that this number will double by the year 2030 (1).

According to reports by the International Diabetes Federation (IDF), more than 8% of the world population (415–420 million people) have diabetes in the year 2015 and the prevalence is expected to rise to 10.4% (642 million) by 2040 (2). The prevalence rate for diabetes mellitus has increased from 4.7% in 1980 to 8.5% in 2014, and its prevalence is increasing rapidly in middle and low-income countries (3). According to the 2006 National Health and Morbidity Survey (NHMS), the prevalence of diabetes in Malaysia is 11.6%, among persons aged 18 years old or older. The 2015 NHMS showed an increase to 17.5% (4). Unfortunately, despite aggressive health awareness campaigns, approximately one in five Malaysians over the age of 30 years old have diabetes (5).

In actuality, diabetes mellitus (DM) increases distress among its patients. Diabetes-related distress (DRD) is defined as an individual’s worries about being diagnosed with diabetes, its medical treatment, emotional stress and support (6). DRD causes somatic symptoms (fatigue, weight loss), smoking behaviour and disease control in adult type 2 diabetes mellitus (T2DM) patients (7). A larger study with longer follow-up demonstrated an association between decreased quality of life with DRD (8). Besides, DRD is closely associated with diabetes-related complications and mortality (6). In addition, among other risk factors for DRD in T2DM patients include poor adherence to the complex therapeutic requirement, insulin initiation, the quality of social support and interpersonal relationship with others including spouses (7). DRD is closely related to the worries of a patient being diagnosed with DM. If not screened and detected at the earlier stage, DRD will eventually lead to severe emotional distress (7).

DRD is closely related to an individual’s moral support, emotional well-being, accessibility to proper diabetes care and ability to manage diabetes (7). Although the prevalence of DRD has been studied in the United States and United Kingdom (9), there is still insufficient studies in Asian countries (7). To address the literature gap, this paper aims to identify the predictors of DRD among diabetic patients in Hospital Universiti Sains Malaysia (USM).

## Methods

This is a cross-sectional study with a convenience sampling method. The recruitment for subjects for this study was started after approval had been granted by Human Research Ethics Committee USM. In addition, informed consent had been obtained from all the participants at the beginning of the study. Among the inclusion criteria for subjects’ recruitment were individuals having T1DM and T2DM diagnosed by a medical doctor, aged 18 years old and above, as well as diabetic patients in both the outpatient and inpatient settings. Meanwhile, the exclusion criteria were those with gestational DM, pediatric patients with diabetes, wheelchair-bound patients or patients in Intensive Care Unit (ICU), patient on regular haemodialysis, individuals diagnosed with a severe psychiatric problem, patients with a history of upper limb injury or deformities with motor impairment, patients with neurological disorders.

### Sample Size

Sample size was calculated using this formula (10):


$$\begin{array}{l} \text{Sample size},n=[{\left(Z\right)}^{2}(p)(1-p)]/{\left(\Delta \right)}^{2}]\\ =[{\left(1.96\right)}^{2}\;(0.49)\;(1-0.49)]/{\left(0.1\right)}^{2}]+20\%\\ =96+20\\ =116\;\text{subjects} \end{array}$$

Where, *n* = sample size, *Z* = value representing the desired confidence level, **Δ** = precision, *p* = anticipated population proportion.

With 80% power of the study, a precision value of 0.1 and a confidence level of 95%, *Z*-score will be 1.96 whereas the prevalence of DRD 49.2% was obtained from the study by Chew et al. (7). They had conducted a study among diabetic patients to determine the prevalence of DRD. By considering a 20% non-response rate, the final number of participants that had been included in this research was about 116 people. However, we only managed to recruit 100 subjects due to the movement control order implemented in March 2020 which hinders data collection.

### Research Parameters and Instrument

Sociodemographic data investigated in this study were age, gender, ethnicity, religion, marital status, educational level, employment status, household income, exercise, smoking status, and duration of diabetes were collected.

Next, anthropometry measurements assessed were weight, height and body mass index (BMI). The weight was measured using a SECA digital weighing scale 803 (SECA Corporation, Germany). Subjects were asked to empty their shirts and trousers pockets as well as remove any additional clothes such as jacket, shawl and coat to obtain more accurate weight measurements. Measurement was taken twice to the nearest kilogram (kg).

Height was measured using the SECA portable stadiometer 206 (SECA GmbH & Co. KG, Hamburg, Germany) to the nearest 0.1 cm. For subjects who were physically unfit to obtain the measurement of weight and height, their weight and height were obtained from the medical folder.

BMI was computed by dividing weight (kg) by height squared (m^2^). The World Health Organization (WHO) BMI cut-off points for Asian population were used for classification as follows: BMI < 18.5 kg/m^2^ (underweight), between 18.5 kg/m^2^ and 22.9 kg/m^2^ (normal), between 23.0 kg/m^2^ and 27.49 kg/m^2^ (overweight) and 27.5 kg/m^2^ or above (obese) (11). For older adults aged 65 years old and above, the cut-off point of > 29 kg/m^2^ is used to classify them as overweight (12).

Lafayette hydraulic hand dynamometer (Lafayette Instrument Company, USA) was used to measure hand grip strength (HGS) with the subject seated with the elbow flexed at 90°, forearm in a neutral position and wrist between 0° and 30° of dorsiflexion (13). The measurement for HGS was taken with the handle set at the second position for all subjects to ensure standardisation. All the subjects were tested on both hands with the right hand first, irrespective of the hand dominance and asked to squeeze the handle as hard as possible. Two measurements of HGS were taken for each hand, with a 20-sec rest between the measurements to reduce fatigue. The mean value of all measurements was then calculated to the nearest kg.

The bioelectrical impedance analysis (BIA) method was used to assess body composition. The equipment used was the Tanita body fat analyser (Tanita Corp, Tokyo, Japan). BIA was used because it was a cheaper method and more convenient to be conducted in both inpatient and outpatient settings. This measurement was repeated twice for the average value. Before testing, subjects were required to adhere to these BIA testing guidelines: i) to not eat or drink 4 h before the test; ii) to maintain normal body hydration; iii) not to consume caffeine or alcohol within 12 h of the test; iv) not to exercise 12 h before the test and v) not to urinate before 30 min of the test (14).

DRD was assessed using the Malay version of the 17-item diabetes distress scale (MDDS-17) (15–16) questionnaire. This questionnaire was designed on a 6-point Likert scale with the lowest score of 1 (not a problem) to the highest score of 6 (very serious problem). MDDS-17 identified problems and difficulties related to diabetes during the past month (15). The scoring for MDDS-17 can either be presented as a total DRD scale score or can be separated into three domains namely the emotional burden (EB), physician-related distress (PD) and therapeutic support. This is slightly different from the original English version of the DDS questionnaire. The English version of the DDS questionnaire has four subdomains, namely the EB, PD, regimen-related distress (RD) and diabetes-related interpersonal distress (ID). For the MDDS-17, the total mean score of less than 2.0 indicated little to no distress, a score between 2.0 and 2.9 indicated moderate distress, and a score of 3.0 or higher represented a high level of distress. The MDDS-17 had high internal consistency (Cronbach’s α = 0.94) (17).

### Statistical Analysis

To analyse the data obtained, the Statistical Package for the Social Sciences (SPSS) version 26.0 (SPSS, Inc., Chicago, IL, USA) was utilised. Next, a normality test was done using the histogram to determine the distribution of the data. Descriptive statistics were employed to present the mean of DRD scores based on domains. In addition, Pearson’s correlation (for normally distributed data) and Spearman’s correlation (for non-normally distributed data) will be employed to determine the correlation between DRD and numerical variables such as age, weight, education years, HGS, body composition and anthropometry. On the other hand, the Kruskal-Wallis test and Mann-Whitney test were used for testing the median differences between non-normally distributed categorical data (exercise, smoking, marital status, gender, household income and duration of diabetes) with DRD scores. Multiple linear regression analysis was performed to determine factors affecting DRD. The model was adjusted for years of education, age and gender. Independent variables for the regression model were selected based on the significant variables from the univariate analysis and previous literature. Finally, multiple linear regression was conducted after the assumptions of linearity, no multicollinearity, independence values of residuals, and homoscedasticity were met. Statistical significance was set at a *P*-value less than 0.05.

## Results

A total of 100 subjects were recruited in this study due to the pandemic situation which was a barrier for patient’s visit to hospital.

### Diabetes-Related Distress

The mean (SD) of total DRD was 1.19 (0.47). The mean score for EB, PD and therapeutic support distress domain were 1.59 (0.67), 1.05 (0.22) and 2.04 (0.71), respectively (Table 1). Prevalence of low DRD was 93% while moderate to high DRD were 6% and 1%, respectively. Since the number of individuals with moderate and high DRD is low, the numerical DRD score was used for analysis.

### Association between Diabetes-Related Distress Score with Sociodemographic, Anthropometry, Body Composition, Lifestyle, Duration of Diabetes and Handgrip Strength

Correlation analysis demonstrated a significant association between education years, BMI, weight, HGS and body fat percentage with DRD score (*P* < 0.05). Subjects with higher education years had weak negative correlation with DRD scores. Similarly, increasing weight, BMI, body fat percentage and HGS had significant weak negative correlation with DRD scores. Higher weight, BMI and body fat percentage contributed to lower DRD scores which indicated lesser diabetic related distress. Furthermore, individuals with better HGS had lower DRD scores (Table 2).

As for the categorical parameters, marital status and exercise were significantly related to DRD scores. Diabetics who were unmarried have a lower DRD score (1.47 [1.53]) as compared to those who are married (1.18 [0.24]) (*P* < 0.05). On the other hand, subjects who never exercise reported having higher DRD scores (*P* < 0.05) (Table 2).

### Predictors of Diabetes-Related Distress among Diabetic Patients in Hospital USM

Table 3 showed the factors affecting DRD among the subjects in this study using multivariate analysis. Multiple linear regression adjusted for age, gender and years of education demonstrated that marital status (single, divorced or widow), and smoking were significant risk factors for DRD score (*P* < 0.05). Marital status had higher impact on the prevalence of DRD than smoking status by comparing the standardised coefficient (0.505 versus 0.229). It was found that an increase in smoking factor significantly increased DRD score by 0.229 unit (95% CI: 0.008, 0.450; *P* = 0.042). Meanwhile, being single, divorced, widow contributed to an expected increase in DRD score by 0.505 units (95% CI: 0.170, 0.84; *P* = 0.004).

## Discussion

In the present study, 93% of our samples experience low DRD, 6% and 1% experience moderate and high DRD, respectively. Our finding was compared with a recent study in Malaysia which revealed that about 49.2% of their T2DM population has moderate distress on a MDDS-17 scale (7). Another study that was done in the USA using the DDS-17 scale showed that 51.3% of the screened participants had moderate to high DRD (18). The distress proportions using DDS-17 in three different studies conducted in Bangladesh, China and Canada were 48.5%, 43%, and 39%, respectively (19–20). On the other hand, two studies from Germany employing the Problem Areas in Diabetes Questionnaire (PAID) demonstrated that 8.9% and 10.7% of their sample experienced distress (21). Contrary to most of the prior studies, our findings of DRD prevalence were very much lower due to convenience sampling and in addition, this discrepancy could also be attributed to the different assessment tools used to assess DRD across different countries and healthcare settings. Apart from that, there were many other variables, including the huge differences between sample size and social-demographic characteristics.

The univariate analysis had shown that low HGS individuals had high DRD scores, but this was not observed in the multivariate regression model. On the other hand, low HGS indicated that the individuals had poor muscle strength. Therefore, HGS had been included as one of the parameters in this study due to the fact that diabetes mellitus was a risk factor of sarcopenia and it had been identified as a possible cause for lower HGS (22). Mild hand muscle weakness could stem from other diabetes complications, such as peripheral neuropathy (23). Similarly, body fat percentage, BMI and weight had significant weak negative univariate correlation with DRD, however these were not observed in the multivariate model. Obese people with diabetics had increased risk of stress due to dysregulation of hypothalamic-pituitary-adrenal (HPA) axis and release of cortisol (24).

Furthermore, this study demonstrated that marital status (single, divorced or widow) was a significant risk factor for DRD score (*P* < 0.05). According to McCaig, marriage can provide motivation and moral support, thereby promoting spouses’ healthy lifestyles (25). Within the closely-knit social network comprising families and communities in the typical Asian customs sufficient social support was probably gathered over time, resulting in less DRD (26).

In line with other previous study (27), the present study found that smoking was significantly associated with DRD. The possible explanation for this finding could be that long-term exposure to nicotine dysregulates the HPA system. Therefore, this causes alterations in the monoamine neurotransmitter system which regulates reactions to stressors (28).

## Strengths and Limitations of Study

To the best of our knowledge, this study helps to identify the factors affecting DRD among diabetic patients in Hospital USM. Apart from that, the objective measurements with standard protocols used to measure HGS and body composition ensured minimum measurement bias. In addition, the questionnaire used in this study has been used and validated in other studies.

However, there are several limitations in this study that are worth noting. First, as this is a cross-sectional study, a causal relationship cannot be established. Besides, the majority of the subjects in Kota Bharu, Kelantan were Malays. Therefore, this sample is not representative of multiple ethnic groups and may limit the generalisability of our findings to the state or country. The COVID-19 pandemic is another barrier for achieving the sample size.

## Conclusion

To conclude, predictors of DRD in this study were smoking and being either single/divorced/widow. Therefore, these factors have to be taken into consideration while receiving medical treatment to ensure more holistic management of the disease and the distress caused by it. A good support system is also essential in reducing the distress faced by the patients. However, the findings from this study have to be confirmed by conducting several larger studies involving multiple study sites around Malaysia.

## Figures and Tables

**Table 1:** DRD based on domains

Domains	Mean (SD)
Emotional burden	1.59 (0.67)
Physician distress	1.05 (0.22)
Therapeutic support	2.04 (0.71)
Total DRD score	1.19 (0.47)

**Table 2:** Association between DRD score and sociodemographic, anthropometry, body composition, lifestyle, duration of diabetes and HGS (*n* = 100)

Parameters	Median (IQR)	*r*-value	*P*-value
Age (years old) a	60.5 (13.8)	−0.018	0.861
Education (years)^a^	11.0 (5.0)	−0.236	0.018*
Weight^a^ (kg)	67.2 (20.7)	−0.246	0.014*
BMI (kg/m^2^)	27.0 (5.7)	−0.266	0.007*
HGS^a^ (kg)	14.8 (10.0)	−0.257	0.010*
Body fat^a^ (%)	27.0 (7.7)	−0.266	0.007*

	**DRD score, median (IQR)**		***P*** **-value**

Gender^b^			
Men	1.18 (0.28)		0.488
Women	1.24 (0.34)		
Marital status^b^			
Married	1.18 (0.24)		0.049*
Single/Divorced/Widow	1.47 (1.53)		
Household income^c^			
< RM,1000	1.32 (0.35)		0.287
RM1,001–RM3,000	1.18 (0.24)		
> RM3,000	1.18 (0.24)		
Smoking status^b^			
Smoker	1.38 (0.35)		0.056
Non-smoker	1.18 (0.26)		
Duration of DM^c^			
< 5 years	1.18 (0.35)		0.386
5–10 years	1.21 (0.24)		
> 10 years	1.24 (0.32)		
Exercise status^c^			
No exercise	1.29 (0.37)		0.023*
≤ 3 times per week	1.18 (0.26)		
> 3 times per week	1.18 (0.18)		

Notes:

a Spearman correlation test was employed;

b Mann-Whitney test;

c Kruskal-Wallis test;

* Results were significant at *P* < 0.05;

IQR = interquartile range

**Table 3:** Risk factors affecting DRD among diabetic patients (*n* = 100)

Variables	B	Standard error	Sig.	95% CI	

				Lower bound	Upper bound
HGS (kg)	−0.013	0.008	0.099	−0.030	0.003
Percentage body fat (%)	0.012	0.018	0.493	−0.023	0.047
Weight	−0.008	0.007	0.234	−0.022	0.005
Single/Divorced/Widow	0.505	0.169	0.004*	0.170	0.841
Smoker	0.229	0.111	0.042*	0.008	0.450

Notes: Dependent variable: DRD score; Model adjusted for age, gender and years of education;

* Significant at *P* < 0.05
